# Evidence of Folliculogenesis and the Potential of Oocyte Recovery from Koalas with Different Levels of Reproductive Pathology

**DOI:** 10.3390/biology14101435

**Published:** 2025-10-17

**Authors:** Stephen D. Johnston, Jackson Boyd, Patricio D. Palacios, Julien Grosmaire, Alexander Lee, Lyndal Hulse, Leslie Vega, Michael Pyne, Andres Gambini, Chiara Palmieri

**Affiliations:** 1 School of Environment, The University of Queensland, Gatton 4343, Australia; j.boyd@uq.edu.au (J.B.); a.e.lee@student.uq.edu.au (A.L.); l.hulse@uq.edu.au (L.H.); 2 School of Veterinary Science, The University of Queensland, Gatton 4343, Australia; a.gambini@uq.edu.au (A.G.); c.palmieri@uq.edu.au (C.P.); 3 School of Agriculture and Food Sustainability, The University of Queensland, Gatton 4343, Australia; p.palaciosbenitez@uq.edu.au; 4 Endeavour Veterinary Ecology, Donnybrook 4510, Australia; julien@endeavorvet.com.au; 5 Currumbin Wildlife Sanctuary, Currumbin 4223, Australia; lvega@cws.org.au (L.V.); m.pyne@cws.org.au (M.P.)

**Keywords:** genetic recovery, ovarian bursitis, reproductive pathology, assisted reproductive pathology, ovarian follicular activity

## Abstract

Each year, hundreds of female koalas are presented to koala hospitals suffering from a range of morbidities, many of which require euthanasia for animal welfare reasons. These koalas represent a possible resource for genetic recovery by means of oocyte retrieval for genome banking or use in assisted reproductive technology. To examine the feasibility of koala oocyte recovery, this study conducted a preliminary survey of follicular activity and disease presence in fixed ovarian tissues from koala cadavers in South East Queensland. Ovarian activity and pathology were assessed by gross examination and histology. Bursal pathology was categorized into koalas with no, small (<10 mm diameter), moderate (10–20 mm diameter), or large (>20 mm diameter) sized bursae, whereas uterine pathology was diagnosed by an experienced reproductive pathologist. Antral follicles were observed in 94.4% of ovaries recovered from koalas with no bursal or uterine pathology (n = 18/44), 95.2% of the ovaries of koalas with bursal but no uterine pathology (n = 11/44), 100% of the ovaries of koalas showing only uterine pathology (n = 4/4) and 89.5% of ovaries from koalas with both bursal and uterine pathology (n = 11/44). Of the fixed ovarian tissue suitable for PCR *Chlamydia* detection (35/44), none were positive. As proof of concept, oocytes were also collected and evaluated from six koala cadavers within 2 h post-mortem. Although further studies are required to determine the quality and viability of the retrieved koala oocytes, our preliminary survey provides strong evidence that ovarian activity mostly continues unabated, irrespective of reproductive pathology, and that oocytes can be recovered successfully.

## Simple Summary

Koalas in eastern Australia are under an increasing threat of extinction from reproductive disease (chlamydiosis) and habitat degradation. We present data on the evidence of ovarian activity and the potential of oocyte collection from ovaries of koala cadavers with varying degrees of reproductive pathology. While we have demonstrated that the majority of koala ovaries show evidence of active folliculogenesis despite varying degrees of reproductive pathology and that oocyte collection from the ovaries of post-mortem koalas is possible, further studies that facilitate in vitro maturation of oocytes leading to transfer of fertilized embryos are now required. This work represents a cautious positive step forward towards the establishment of frozen genome bank for the koala.

## 1. Introduction

Recently listed as endangered by the governments of Queensland, New South Wales, and the Australian Capital Territory, the koala faces multiple anthropogenic threats, the most significant of which are habitat degradation and disease [1]. Chlamydiosis is considered one of the most significant threats to wild koalas and is responsible for recent declines in their population density across eastern Australia [2,3]. The bacterium *Chlamydia pecorum* is the primary infectious agent and is the most prevalent sexually transmitted bacterial disease known in koalas [4]; it causes severe pathology of the female reproductive tract, potentially leading to infertility associated with ovarian bursitis, salpingitis, metritis, and/or vaginitis [5,6].

While *C. pecorum* has pathological effects on the female reproductive tract, studies to date suggest that the ovaries appear to be largely unaffected, or at the very least, show no evidence of gross pathology, irrespective of the pathology of their surrounding bursa [7,8]. Ovaries with cystic dilation of the ovarian bursae typically show evidence of normal follicular and luteal activity and may contain fluid-filled follicles, corpora lutea (CLs), corpora haemorrhagica or corpora albicans; consequently, ovaries of infected koalas may still be functional despite the presence of severe cystic bursal lesions, thus producing estrogen secreting pre-ovulatory follicles which can still potentially induce estrous behavior in females; this may pose a significant threat to koala populations in terms of chlamydial epidemiology, as infected females may remain sexually receptive and thereby serve to further transmit *Chlamydia* to other sexual partners. Treatment for *C. pecorum* is currently limited to antibiotics; however, this approach has significant restrictions, as koalas have a highly sensitive gastrointestinal microbiome [3]. Since treatment of *Chlamydia* is difficult and has risks, especially for individuals not in captive care, we propose the use of gamete rescue technology combined with the use of artificial reproductive technology as a means of rescuing the genetic diversity of koala populations that would otherwise be lost.

Research on marsupial oocyte collection post-mortem, in vitro maturation (IVM), and subsequent embryo culture remains limited and uneven, with most foundational studies dating from the 1970s–1990s and focused on a few model species such as the tammar wallaby (*Notamacropus eugenii*), brushtail possum (*Trichosurus vulpecula*), and gray short-tailed opossum (*Monodelphis domestica*). Systematic evaluations of post-mortem oocyte recovery are particularly scarce, though this approach represents a valuable opportunity to recover gametes from euthanised or deceased wildlife. Early embryological studies established cleavage and blastocyst timing [9] and confirmed the feasibility of in vitro fertilization (IVF) [10] in *Monodelphis domestica* and intracytoplasmic sperm injection (ICSI) of tammar wallaby oocytes [11,12]. Subsequent work expanded these findings to the bare-nosed wombat (*Vombatus ursinus*) [13] and fat-tailed dunnart (*Sminthopsis crassicaudata*) [14], showing that although fertilization and early cleavage can be achieved, no live births have yet been reported following ICSI in marsupials. Howell and Witt [15] have also highlighted that robust, species-specific protocols for oocyte IVM and embryo culture remain absent for nearly all marsupials. Recent proof-of-concept studies from our group demonstrate that oocyte collection followed by in vitro maturation and ICSI allow early embryo production in macropods [16], underscoring both the feasibility of marsupial assisted reproduction and the urgent need to optimize oocyte recovery and maturation protocols for broader conservation applications. Oocytes recovered from post-mortem koalas or following surgical ovariohysterectomy have the potential to be used as gametes in assisted reproductive technology, such as the production of embryos by means of in vitro fertilization (IVF), intracytoplasmic sperm injection (ICSI), and establishment of frozen genome banks [17]. The present study surveyed the gross and histological appearance of fixed ovaries from koalas presented to two wildlife veterinary practices in South East Queensland in 2023 and 2024 to determine evidence of folliculogenesis and the potential for oocyte recovery from koalas with varying levels of reproductive disease. Ovaries from koalas with various morbidities (e.g., trauma, reproductive pathology, ill-thrift) and which subsequently required euthanasia because of animal welfare reasons, or which underwent ovariohysterectomy surgery [18] were examined. We were particularly interested in examining ovaries from animals with bursal and uterine pathology. Freshly collected ovaries were also recovered from 6 koalas euthanized due to trauma injury as a proof of concept for oocyte recovery in this species.

## 2. Materials and Methods

### 2.1. Animals

This study examined reproductive tissue from a total of 44 female koalas presented to two koala hospitals in South East Queensland [Endeavour Veterinary Ecology (EVE; n = 38) and Currumbin Wildlife Hospital (CWH; n = 6)]. Reproductive tracts were either recovered from animals following an ovariohysterectomy procedure as part of their veterinary treatment for reproductive-related pathology or from koalas that were euthanized on welfare grounds (e.g., trauma, septicemia, lymphoma, or ill-thrift) or died in the wild and promptly recovered. Thirty-seven koalas examined in this study were part of a koala tracking and monitoring program undertaken by EVE in Queensland and northern New South Wales (NSW), with 32 originating from the Coomera Connector Koala Tagging and Monitoring Program in the South East Queensland bioregion, Sunshine Coast-Gold Coast Lowlands, and Burringbar Conondale Ranges subregions. Four koalas originated from the Brigalow Belt North bioregion, Isaac-Comet Downs subregion, and two koalas from the NSW North Coast bioregion, Barrington subregion. Based on tooth wear estimates [19], koalas from EVE had an age range of 2 to 11 years, with a mean age of 5 years. Koalas admitted to CWH had a mean age of 4 years old and were all rescued from the Gold Coast region, with the exception of one from the Scenic Rim region. 

### 2.2. Clinical Diagnosis

Diagnosis involved a thorough veterinary clinical examination, ultrasonography of the urinary and reproductive tracts [20], and LAMP testing for *Chlamydia pecorum* [21]. Ultrasonography was primarily used to diagnose reproductive diseases associated with enlarged ovarian bursa or distended uteri; however, Doppler ultrasonography, fluid analysis, and cytology were also used to detect smaller-sized cysts and to identify the presence of peritoneal and purulent fluid in order to distinguish normative and pathological changes. Female koalas assessed for euthanasia because of animal welfare reasons were injected with a lethal dose of pentobarbitone [22], and the reproductive tract was recovered and fixed in 10% neutral buffered formalin.

For koalas undergoing ovariohysterectomy as a treatment for reproductive pathology, surgery [18] was performed by experienced koala veterinarians. Under gaseous isoflurane anesthesia, a lower abdomen midline laparotomy was performed to expose the reproductive tract. Once identified, large cysts were drained using a 16-G needle and 10 mL syringe, after which a combination of sharp and blunt dissection was used to break down adhesions to the abdominal wall and surrounding organs. A surgical dissection window was made in the broad ligament using hemostats before using a 2 or 3-clamp technique to ligate ovarian vessels bilaterally. The uteri were then ligated adjacent to the cervices using a 2-clamp technique, and the stump was ligated with a transfixing ligature before closing the abdomen. Immediately upon removal from the abdomen, the reproductive tract was placed in 10% neutral buffered formalin.

Once the tissue samples arrived at UQ, they were initially examined for gross pathology. Each reproductive tract was photographed and carefully inspected for cystic lesions on the surface of the ovarian bursae and uterine pathology by an experienced reproductive veterinary pathologist (CP). The gross pathology assessment in this study was primarily based on the cystic dilation of the ovarian bursa (or bursal dilation), as this is the most common and characteristic pathological symptom of chlamydial infection in female koalas. For cases with cystic dilation, the size of the ovarian bursa was estimated and broadly categorized as either mild (<10 mm diameter), moderate (10–20 mm diameter), or severe (>20 mm diameter), and confirmed with previously recorded clinical data. Significant pathologies of the uterine tissue were also noted, including evidence of hydrosalpinx, hydrometra, and pyometra, and whether the pathology was either bilateral or unilateral. Of the 44 koalas examined for the presence of ovarian activity in this study, a total of 18 showed no bursal or uterine pathology but were euthanized for other animal welfare reasons, 11 females had bursal pathology only (4 bilateral and 7 unilateral), 4 had uterine pathology only (2 bilateral and 2 unilateral) and 11 had coincident bursal and uterine pathology (8 bilateral and 3 unilateral).

### 2.3. Gross Observations and Histology of the Ovary

Each ovary was dissected free of its bursa and photographed for subsequent identification of ovarian activity (antral follicles, corpora lutea, or corpora albicans). Both ovaries were then prepared for standard histopathology. Once embedded in paraffin, an automatic microtome (Leica RM2255, Victoria, Australia) was used to section the blocks to 4 μm. A representative sagittal section deep within the tissue was stained with haematoxylin and eosin (HE) using an auto stainer (Leica ST5020, Victoria, Australia). Ovarian sections were then photographed using a slide scanner (Olympus Evident Scientific Slideview VS200, Victoria, Australia) and images viewed using the associated scanner software (Olympus Evident Scientific OlyVIA V4.2).

### 2.4. Assessment of Ovarian Activity

Reproductive tracts were examined using the ovary as the primary experimental unit; however, in some cases, only one ovary per koala was available for analysis. Based on gross morphology and histology, each koala ovary was assessed for the presence of antral follicles (2–3 mm diameter) or other ovarian structures as defined by Pagliarani et al. [23], including preovulatory follicles (4–7 mm diameter), corpora lutea (active ≥ 5 mm) or corpora albicans (regressing CLs 2–4 mm). Ovarian analyses also focused on the presence and severity of bursal pathology (classified as mild, moderate, and severe) and any uterine pathology.

### 2.5. Chlamydia Pecorum PCR

To test for the presence of *Chlamydia pecorum* DNA in the koala ovaries, 4 × tissue sections were cut at 20 μm from each formalin-fixed paraffin-embedded (FFPE) block and transferred into 1.5 mL microcentrifuge tubes. Deparaffinization was performed by adding 1 mL of xylene, vortexing briefly, and incubating the samples at room temperature for 10 min. The samples were then centrifuged at 14,100× *g* (14,500 rpm) for 5 min, and the supernatant was discarded. This xylene wash was repeated once. Rehydration was then carried out through a graded ethanol series by sequential washing with 1 mL of 100%, 95%, 70%, and 50% ethanol, each step followed by vortexing, centrifugation, and removal of the supernatant. After the final ethanol wash, the tissue pellets were air-dried at room temperature to remove residual ethanol. DNA extraction was performed using the Promega ReliaPrep^TM^ FFPE gDNA Miniprep System DNA extraction kit as per the manufacturer’s instructions (Promega, catalog number A2351, Mannheim, Germany). Sections from both ovaries were processed together and then analyzed for the presence of *Chlamydia pecorum* DNA using the multiplex real-time PCR assay as fully described in Hulse et al. [24]. The oligonucleotide sequence of primers and fluorogenic probes for 2 multiplex real-time PCR panels used to detect *Chlamydia pecorum* and other pathogens is provided in Supplementary Figure S1 and Table S1, in addition to the positive cutoff CT value. Of the 44 koalas examined, koala *β*-actin from the fixed tissue sections was only detected in 35 koalas.

### 2.6. Recovery of Fresh Oocytes

**Figure 1:**
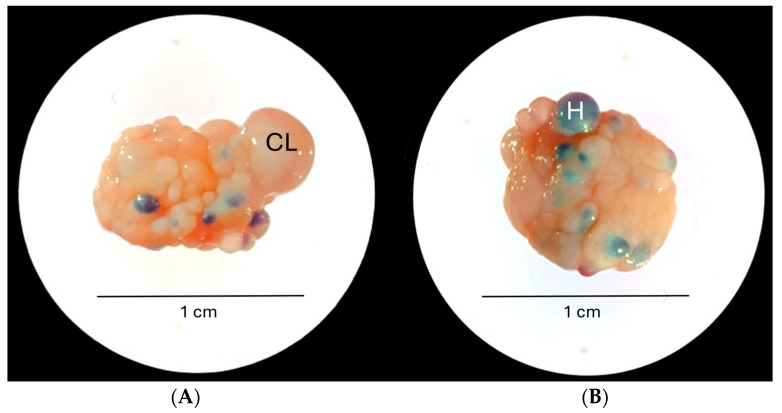
Left (**A**) and right (**B**) ovaries of a female koala immediately following abdominal dissection and removal of the bursae. CL—regressing CL; H—haemorrhagic follicle.

As a prelude to future studies and proof of concept, oocyte extraction from the ovaries of six freshly euthanized trauma koalas was also attempted. Ovaries were retrieved via abdominal incision approximately 2 h after euthanasia and placed in 15 mL Falcon tubes (Corning^®^-CLS430791, Corning, Glendale, AZ, USA) containing room temperature Dulbecco’s phosphate-buffered saline (DPBS; Gibco^^®^^, 14190-136, Waltham, MA, USA) supplemented with 1% (*v*/*v*) antibiotic-antimycotic (Gibco^®^, 15240) and transported for 1 h at ~22 °C in a Styrofoam box to the laboratory. Ovaries did not present any obvious alterations associated with Chlamydia infection. Upon arrival, ovaries were transferred to a 60 mm TC-treated Culture Dish (Corning^®^-430166) containing pre-warmed DPBS (35.5 °C) supplemented with 1% antibiotic-antimycotic and maintained on a heated table (OkoLab H401, Ottaviano, Italy) at the same temperature. To expose the follicles, the bursae, blood vessels, and adjacent connective tissue were removed (Figure 1). Follicles were collapsed by puncturing the ovary with a 27-G needle, followed by superficial incisions across the ovarian surface using a scalpel blade to release the oocytes. The resulting suspensions were examined under a stereomicroscope (SMZ800N, Nikon Corporation, Tokyo, Japan) on a heated table set at 35 °C to evaluate oocyte recovery. Images of retrieved cells were captured using an inverted microscope (Nikon Eclipse Ti2-A, Nikon Corporation, Tokyo, Japan) with RI Viewer Imaging Software (Version 2.4.1, Research Instruments Pte Ltd., Singapore). Following imaging, oocytes were broadly classified as (A) cumulus–oocyte complexes (COCs), (B) oocytes lacking surrounding cumulus cells but with apparently intact zona pellucida and oolemma, and (C) degenerated oocytes exhibiting oocyte membrane rupture and damaged zona pellucida. The oocytes were then fixed and stored for future research projects beyond the scope of the current methodology.

## 3. Results

### 3.1. Evidence of Follicular Development

Table 1 reports the percentage of ovaries from which oocytes were recovered from koalas with differing degrees of reproductive pathology. Table 2, Table 3, Table 4 and Table 5 summarize the individual koala findings of ovarian activity of both ovaries with respect to the incidence of bursal and uterine pathology. 

Table 2 reports on the ovaries that contained antral follicles from individual koalas with no evidence of bursal or uterine pathology. This cohort consisted of koalas with a broad range of morbidities that included various neoplasia, cystitis, hepatitis, peritonitis, osteomyelitis, septicemia, typhlocolitis, urolithiasis, severe tick burden, and physical trauma. Two koalas (K32 and K39) had one ovary in which no antral follicles were apparent, and one of these (K39) had an unusually high proportion of interstitial tissue in the ovarian stroma. Both ovaries of K18 showed evidence of ovarian carcinoma arising from the surface epithelium and invading the bursa. Of 26 out of 36 (72.2%) koala ovaries for which koala *β*-actin was detected by PCR, none were positive for *Chlamydia pecorum*. Table 2 also indicates that 9 of the 18 (50.0%) koalas showed evidence of ovarian activity consistent with a follicle reaching a pre-ovulatory size, or an active or regressing CL; all reproductive tracts of these koalas were recovered within the recognized breeding period of the koala in South East Queensland (September–April).

Table 3 reports on the ovaries that contained antral follicles from individual koalas with evidence of bursal pathology but no uterine pathology. Of the 22 koala ovaries examined in this category, 21 (95.2%) had antral follicles that potentially could be recovered for oocyte maturation; in all but one animal (K20), both ovaries had antral follicles. One of the ovaries of K20 also showed evidence of severe fibrosis and contained an abscess. Antral follicles were mostly present on the ovaries, irrespective of the size of the bursal lesion. Of the 18 out of 22 (81.8%) koala ovaries for which koala B-actin was detected by PCR, none were positive for *Chlamydia pecorum*. Eight of the eleven (72.7%) females in this category also presented with active or regressing corpora lutea; evidence that these females had cycled in the near term.

Table 4 reports ovaries that contained antral follicles from koalas with no evidence of bursal pathology, but which had some degree of uterine pathology. All eight ovaries (100%) examined from koalas in this category had antral follicles, and none were PCR positive for *Chlamydia pecorum*. Both uteri of K12 were found to have endometritis, K24 had a unilateral pyometra which contained 20 mL of purulent fluid and uterine adhesions to the body wall and ovarian vasculature, and K31 presented with a unilateral hydrometra. All four koalas appeared to be reproductively active; the ovaries of K12, K24, and K31 possessed large active corpora lutea, and K37 was observed with a presumptive pre-ovulatory follicle.

Table 5 reports ovaries that contained antral follicles from koalas with both bursal and uterine pathology. Of the 19 ovaries examined, 17/19 (89.5%) in this category had antral follicles that could potentially be recovered, although in three animals (K1, K10, and K22), one of the two ovaries was not recovered or lost in dissection or processing. Of the 18 out of 22 (81.8%) koala ovaries for which koala *β*-actin was detected by PCR, none were positive for *Chlamydia pecorum*. This cohort had bursal cysts ranging from mild to severe on both or one ovary and uterine pathology ranging from non-descript dilated uteri to definitive endometritis, pyometra, and hydrometra. Ovaries of K1, K33, and K41 possessed large active corpora lutea, whereas the ovaries of K22, K34, and K35 were observed with large presumptive pre-ovulatory follicles. The only ovary of K10 that was recovered showed no evidence of antral follicles, and this was also the case for one of the ovaries of K38; both ovaries could be regarded as being in an anestrus state.

### 3.2. Bursal and Uterine Adhesions

Although not anticipated as part of the original data analysis, it also became obvious that bursal and uterine adhesions were often grossly observed by the clinician, such that a secondary analysis of their occurrence (where data were present) was investigated with respect to the number of cases, noting adhesions or the lack of adhesions. Bursal and/or uterine adhesions were detected in 4 of 18 (22.2%) koalas with no bursal or uterine pathology, 8 of 11 (72.7%) koalas with bursal pathology but no uterine pathology, 2 of 4 (50%) koalas with no bursal pathology but with uterine pathology and 2 of 11 (18.2%) koalas with both bursal and uterine pathology.

### 3.3. Preliminary Study of Oocyte Recovery from Freshly Collected Ovaries

**Figure 2:**
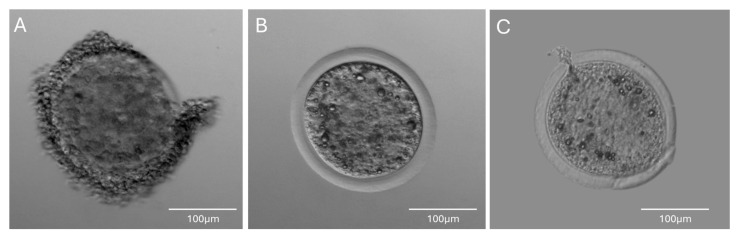
Koala oocyte classification after retrieval: (**A**) Cumulus–oocyte complex (COCs), (**B**) Oocytes lacking surrounding cumulus cells but with apparently intact zona pellucida and oolemma, and (**C**) degenerated oocyte exhibiting oocyte membrane rupture and damaged zona pellucida.

From six female koalas euthanized because of trauma, a total of 120 immature oocytes were recovered from 12 ovaries. This corresponded to an average oocyte recovery of 20.0 ± 3.9 (mean ± SEM; Range 10–36) oocytes per female. Upon dissection, oocytes were classified into three categories: (A) cumulus–oocyte complexes (COCs), (B) oocytes lacking COCs but with intact membranes, and (C) degenerated oocytes exhibiting membrane or zona rupture (see Figure 2). Of the total oocytes recovered, 90 (75%) were categorized as category A, 11 (9.2%) as B, and 19 (15.8%) as group C. 

## 4. Discussion

As nearly all ovaries (approximately 95%) examined in our study exhibited antral follicles, our findings have demonstrated that active folliculogenesis occurred in most koala ovaries, regardless of the presence of bursal or uterine reproductive pathology. Consequently, our preliminary observations suggest that there was no clear relationship between the presence of antral follicles and that of bursal or uterine pathology, although koalas that possessed both bursal and uterine pathology showed the lowest number of ovaries with antral follicles. As a proof of concept, for the first time, we also recovered 120 immature oocytes of varying quality from a total of 12 ovaries collected from six euthanized koalas. However, further studies are required to determine the viability of these cells and the importance and necessity of intact cumulus cells for successful in vitro maturation.

Of the 44 formalin-fixed ovaries processed, koala *β-actin* was successfully amplified in 35, indicating that DNA quality was variably preserved across samples. This inconsistency is likely attributable to differences in tissue quality, fixation conditions, and DNA fragmentation introduced during histological processing, which may have reduced the amount of amplifiable template in some cases. In the subset of 35 ovaries where *β-actin* was detected, none were PCR positive for *C. pecorum* DNA. Because *β-actin* was successfully amplified, this suggests that total DNA integrity was maintained in those samples despite fixation and histological processing. Therefore, the absence of *C. pecorum* DNA more likely reflects a true lack of detectable infection in the ovarian tissue, rather than DNA degradation. However, future attempts of oocyte recovery will include *Chlamydia* PCR testing of freshly obtained ovarian tissue post-retrieval of the oocytes, but these initial results suggest that oocytes are likely to be free of chlamydial organisms and therefore suitable for genome banking.

Our findings represent a cautious first step towards the production of embryos and the possibility of cryo-banking female koala genetic material. Given that koala spermatozoa have proven difficult to cryopreserve [17], the production of embryos by means of procedures such as ICSI may represent an alternative artificial reproductive technique for the species, which is further supported by our recent success in the production of an Eastern Grey Kangaroo embryo following sperm injection into an in vitro mature oocyte [16]. Future studies should document koala oocyte morphometrics, detection of the oocyte cytoskeleton by means of actin and tubulin immunofluorescence staining, and the use of RNA-seq and transcriptomics to understand gene expression at different stages of oocyte maturation.

The successful recovery of koala oocytes will also facilitate new lines of research possibilities, leading to a better understanding of fertilization biology in this species. There are currently large gaps in our knowledge regards the in vitro maturation of marsupial oocytes, in terms of the role of the granulosa cells and the importance of the correct hormonal milieu needed in the media. Although ICSI production of embryos would represent a significant advance in our knowledge and technical expertise, there is still the issue of understanding the importance of the mucoid coat and shell membrane to marsupial embryogenesis [25] and which is likely to be difficult to replicate in the laboratory; one approach that avoids this problem would be to transfer ICSI derived embryos directly into the recipient oviduct.

An understanding of acrosome reaction is extremely limited for marsupials, in general, and represents a particular challenge in the Vombatiforms (Koalas and Wombats) as these species possess an acrosome that is located along the inner curvature of the sperm head, such that the physical mechanics of fertilization remain elusive. Oocytes recovered from koala ovaries can now be used to investigate how the sperm cell might be capable of penetrating the unusually thickened zona pellucida of the koala and wombat [23,26].

From a clinical perspective, the reproductive tracts of the koalas examined in this study, and from our previous research [23], suggest that care should be taken when making a veterinary diagnosis of reproductive pathology, as the tissue of the koala reproductive tract (oviduct, uterus, vaginal cul-de-sac, lateral vaginae and urogenital sinus) is particularly sensitive to the stage of the reproductive cycle. A pre-ovulatory follicle producing systemic oestradiol-17*β* or a corpus luteum secreting progesterone in the bloodstream has a profound effect on the proliferation of normal reproductive tissues [23], and therefore, could present as a misdiagnosis of reproductive pathology during ultrasonographic examination. We were also surprised by the widespread occurrence of reproductive tract adhesions in koalas with and without gross reproductive pathology; the pathogenesis of these adhesions and their impact on female fertility require further investigation.

## 5. Conclusions

While this study has demonstrated active folliculogenesis of formalin fixed ovaries of post-mortem koalas with and without reproductive pathology and the recovery of oocytes from freshly collected ovaries, the next steps will require further investigations and research that assesses the quality of the koala oocyte upon recovery, the establishment of suitable media and in vitro conditions that promote koala oocyte in vitro maturation, disease testing of koala gametes, the production of ICSI fertilized koala embryos, and the development of methods that support early embryo culture and transfer into synchronized recipient koalas.

## Figures and Tables

**Table 1:** Percentage of ovaries recovered from koalas with reproductive pathology that possessed antral follicles.

Reproductive Pathology (n = 44 Koalas; 85 Ovaries)	% of Ovaries with Antral Follicles
No gross pathology (n = 18 koalas; 36 ovaries)	94.4
Bursal pathology only (n = 11 koalas; 22 ovaries)	95.2
Uterine pathology only (n = 4 koalas; 8 ovaries)	100
Bursal and uterine pathology (n = 11 koalas; 19 ovaries)	90.1

**Table 2:** Individual koala ovarian activity with no evidence of bursal and uterine pathology.

Ovary ID	Antral Follicles	Comments	Ovary ID	Antral Follicles	Comments
K2a	Yes	-	K2b	Yes	-
K11a	Yes	-	K11b	Yes	-
K13a	Yes	Presumptive preovulatory follicle 3.6mm diameter	K13b	Yes	-
K14a	Yes	Degenerating CL 3.3 mm diameter	K14b	Yes	-
K15a	Yes	Degenerating CL 3.5 mm diameter	K15b	Yes	-
K18a	Yes	Ovarian carcinoma	K18b	Yes	Ovarian carcinoma
K19a	Yes	Active CL 5.1 mm diameter + smaller regressing CL 2.5 mm diameter	K19b	Yes	2 small regressing CLs, 2.8 mm and 3 mm in diameter
K23a	Yes	-	K23b	Yes	-
K25a	Yes	Regressing CL 4.4 mm diameter	K25b	Yes	-
K26a	Yes	Regressing CL 3.2 mm diameter	K26b	Yes	-
K27a	Yes	-	K27b	Yes	-
K28a	Yes	Bursal–ovarian adhesions; Regressing CL 2.5 mm diameter	K28b	Yes	-
K29a	Yes	Few antral follicles; high proportion of interstitial tissue	K29b	Yes	Few antral follicles; high proportion of interstitial tissue
K32a	Yes	Bursal-ovarian adhesions	K32b	No	-
K39a	Yes	High proportion of interstitial tissue	K39b	No	High proportion of interstitial tissue
K40a	Yes	Moderate CL 4 mm diameter	K40b	Yes	-
K42a	Yes	Large preovulatory follicle 3.9 mm diameter	K42b	Yes	Regressing CL 3.5 mm diameter
K43a	Yes	-	K43b	Yes	-

**Table 3:** Individual koala ovarian activity with evidence of bursal but no uterine pathology.

Ovary ID	Bursal Pathology	Antral Follicles	Comments	Ovary ID	Bursal Pathology	Antral Follicles	Comments
K4a	Moderate	Yes	-	K4b	Severe	Yes	Active CL 7.3 mm diameter
K5a	Moderate	Yes		K5b	Normal	Yes	Active CL 5.7 mm diameter
K7a	Moderate	Yes	-	K7b	Normal	Yes	Regressing CL 2.7 mm diameter
K8a	Severe	Yes	Bursal–ovarian adhesions	K8b	Severe	Yes	
K9a	Moderate	Yes	Regressing CL 3.6 mm diameter	K9b	Normal	Yes	-
K16a	Moderate	Yes	-	K16b	Normal	Yes	-
K17a	Mild	Yes	-	K17b	Mild	Yes	-
K20a	Moderate	No	Inflammation and fibrosis in the ovary; abscess	K20b	Normal	Yes	
K30a	Moderate	Yes	-	K30b	Normal	Yes	
K44a	Severe	Yes	Inflammation of the bursa	K44b	Normal	Yes	Moderate CL 4.0 mm diameter
K45a	Mild	Yes	Regressing CL 3.0 mm diameter	K45b	Mild	Yes	Regressing CL 2.8 mm diameter

**Table 4:** Individual koala ovarian activity with evidence of uterine but no bursal pathology.

Ovary ID	Uterine Pathology	Antral Follicles	Comments	Ovary ID	Uterine Pathology	Antral Follicles	Comments
K12a	Pathology	Yes	Endometritis; Active CL 5.6 mm diameter	K12b	Pathology	Yes	Endometritis
K24a	Normal	Yes	Active CL 5.4 mm diameter	K24b	Pathology	Yes	Pyometra; Regressing CL 3.4 mm diameter
K31a	Pathology	Yes	Hydrometra; Active CL 7 mm diameter, Regressing CLs 3.2 mm and 4.6 mm diameter	K31b	Normal	Yes	Regressing CL 4.6 mm diameter
K37a	Pathology	Yes	Presumptive preovulatory follicle 3.9 mm diameter	K37b	Pathology	Yes	Uterine cyst

**Table 5:** Individual koala ovarian activity with evidence of bursal and uterine pathology.

Ovary ID	Bursal Pathology	Uterine Pathology	Antral Follicles	Comments	Ovary ID	Bursal Pathology	Uterine Pathology	Antral Follicles	Comments
K1a	Severe	Pathology	Yes	Pyometra; Endometritis; Regressing CL 2.4 mm diameter	K1b	Normal	Normal	N/A	Ovary not found in the bursa
K3a	Mild	Pathology	Yes	Hydrometra	K3b	Normal	Pathology	Yes	Hydrometra
K6a	Normal	Pathology	Yes	Thrombosis of the arteries on the ovarian surface	K6b	Mild	Normal	Yes	-
K10a	Moderate	Normal	No	Anestrus, bursal-ovarian adhesions	K10b	Moderate	Normal	N/A	Ovary not found in the bursa
K22a	Normal	Pathology	Yes	Hydrometra; Presumptive preovulatory follicle 5.3 mm diameter	K22b	Normal	Normal	N/A	Ovary not found in the bursa
K33a	Mild	Pathology	Yes	Regressing CL 4.0 mm diameter	K33b	Normal	Pathology	Yes	Active CL 5.8 mm diameter
K34a	Mild	Pathology	Yes	Dilated uterus	K34b	Mild	Normal	Yes	Presumptive preovulatory follicle 4.5 mm diameter
K35a	Moderate	Pathology	Yes	Presumptive preovulatory follicle 5.9 mm diameter	K35b	Moderate	Pathology	Yes	-
K36a	Moderate	Pathology	Yes	Pyometra	K36b	Moderate	Normal	Yes	-
K38a	Normal	Pathology	Yes	Dilated uterus; Bursal-ovarian adhesions; Presumptive preovulatory 5.2 mm diameter	K38b	Severe	Pathology	No	Dilated uterus; Bursal-ovarian adhesions
K41a	Mild	Pathology	Yes		K41b	Normal	Normal	Yes	Active CL 5.1 mm diameter

## Data Availability

The data presented in this study are available on request from the corresponding author due to privacy reasons.

## Supplementary Materials

The following supporting information can be downloaded at: https://www.mdpi.com/article/10.3390/biology14101435/s1, Figure S1: Representations of different levels of the gross bursal and uterine pathology grading scale. A-C: no bursal or uterine pathology–bursa dissected open; D: small bursal cystic dilation–bursa dissected open; E: moderate bursal cystic dilation—bursa closed; F: moderate bursal cystic dilation-bursa dissected open and enlarged unilateral pus filled uterus; G: large bursal cystic dilation and enlarged unilateral uterus; H: small bursal cystic dilation with enlarged unilateral uteri. Bl–bladder, Bu–ovarian bursa, Ce–Cervix, Cl–corpus luteum, In–infundibulum, Od–oviduct, Ov–ovary, Po–preovulatory follicle, Ut–uterus. Table S1: Oligonucleotide sequence of primers and fluorogenic probes for 2 multiplex real-time PCR panels.
