# Supplementary material for: Evidence of Folliculogenesis and the Potential of Oocyte Recovery from Koalas with Different Levels of Reproductive Pathology
Source: Biology (Basel). 2025 Oct 17;14(10):1435. doi: 10.3390/biology14101435 (PMC12561298; doi:10.3390/biology14101435)
Supplement: Supplementary file 1 [file biology-14-01435-s001.zip › Supplementary Figure S1.pdf]

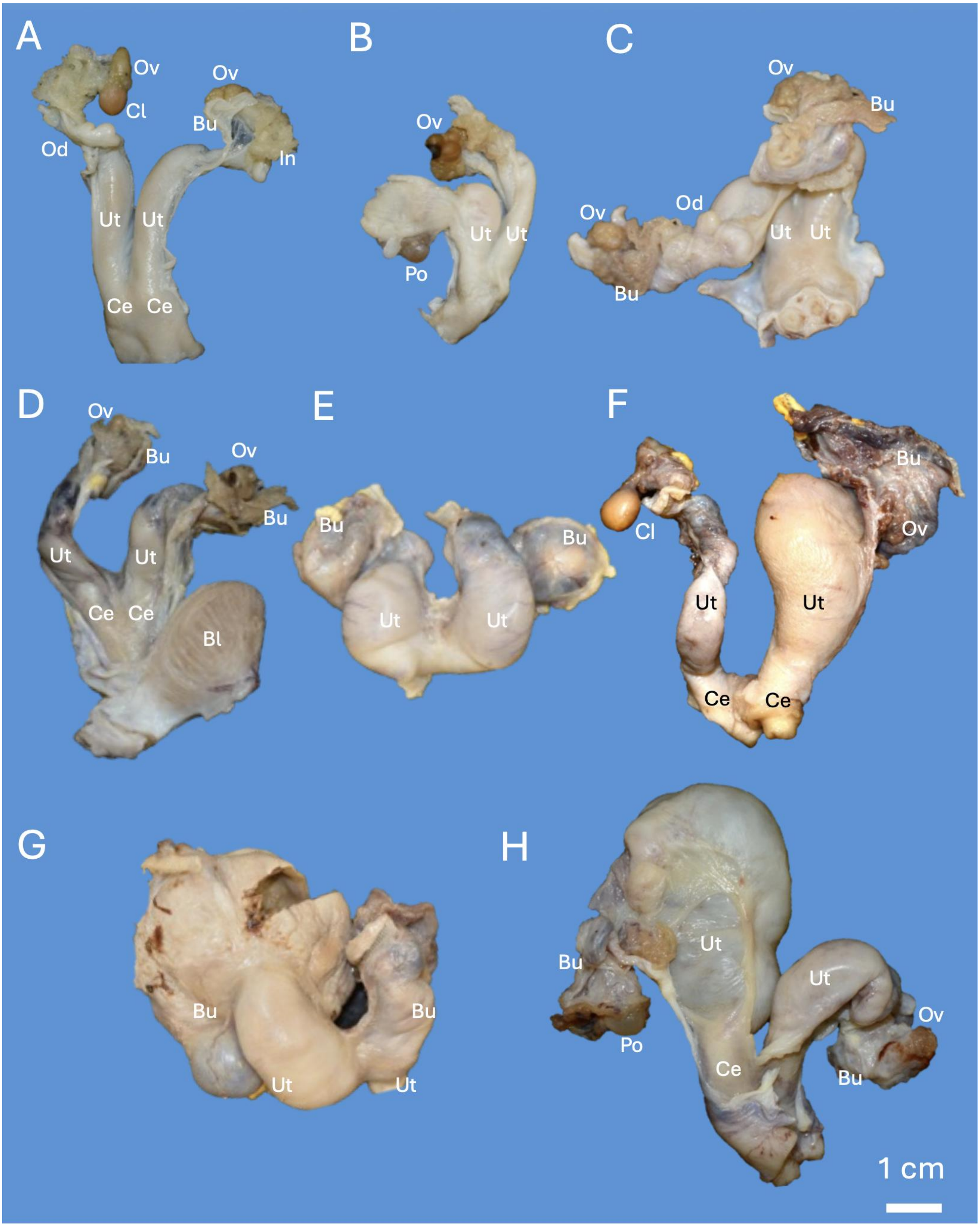

Figure S1: Representations of different levels of the gross bursal and uterine pathology grading scale. A - C: no bursal or uterine pathology – bursa dissected open; D: small bursal cystic dilation – bursa dissected open; E: moderate bursal cystic dilation – bursa closed; F: moderate bursal cystic dilation - bursa dissected open and enlarged unilateral pus filled uterus; G: large bursal cystic dilation and enlarged unilateral uterus; H: small bursal cystic dilation with enlarged unilateral uteri. Bl – bladder, Bu – ovarian bursa, Ce – Cervix, Cl – corpus luteum, In – infundibulum, Od – oviduct, Ov – ovary, Po – preovulatory follicle, Ut – uterus.
