# Supplementary material for: Evidence of Folliculogenesis and the Potential of Oocyte Recovery from Koalas with Different Levels of Reproductive Pathology
Source: Biology (Basel). 2025 Oct 17;14(10):1435. doi: 10.3390/biology14101435 (PMC12561298; doi:10.3390/biology14101435)
Supplement: Supplementary file 1 [file biology-14-01435-s001.zip › Supplementary Table S1.pdf]

Table S1. Oligonucleotide sequence of primers and fluorogenic probes for 2 multiplex real-time PCR panels.

Target gene/Primer	Primer or probe sequence (5'–3')	Ct to Positivity
**Multiplex 1**		
Phascolarctos cinereus beta-actin mRNA		
BetaActin-F	CTCAGATTATGTTTGAGACCTTC	≤35
BetaActin-R	CCTTCATAGATGGGCACA	
BetaActin-Cy5	Cy5-ACCATCACCAGAGTCCATCACAAT	
23S rRNA *Chlamydia*		
LH23S-F	GCTCACCAATCGAGAATC	≤35
LH23S-R	CCAACACTCCTTTCGGTA	
LH23S-Cy5.5	Cy5.5-CTGAATACTACGCTCTCCTACCGC	
16S rRNA *Mycoplasma*		
MycSp-F	GGTACAAAGAGACGCAATA	≤35
MycSp-R	GCGATTACTAGCGATTCC	
MycSp-VIC	VIC-CGAATAGCAGACTTCAATCCGAACT	
16S rRNA *Ureaplasma*		
UreaSp-F	CGTGAGATGTTGGGTTAA	≤35
UreaSp-R	AAGGGGCATGATGATTTG	
UreaSp-FAM	FAM-CCACCTTCCTCTACCTTGCGG	
**Multiplex 2**		
*omp*B of *Chlamydia pecorum*		
CPecOmpB-F	CCAAGCATAATCGTAACAA	≤35
CPecOmpB-R	CGAAGCAAGATTCTTGTC	
CPecOmpB-Cy5	Cy5-ACTTGTTGGCAATTCTTCTCTTCACA	
*omp*B of *Chlamydia pneumoniae*		
CPneOmpB-F	CAGAGTCTCTGATTACTAAGA	≤35
CPneOmpB-R	ACTGGTTGTTTATTTCTACG	
CPneOmpB-VIC	VIC-CAAGTCTAACCTTCTTCGCTGTCATAG	
*fla*A-*fli*A intergenic spacer of *Bordetella bronchiseptica*		
BordSp-F	GCACATTTCCGAACTTCA	≤35
BordSp-R	CCATCTGTAACATCGGAC	
BordSp-FAM	FAM-TTAAGTCCGTCGCAAACCTGC	
